# Electronic implementation dataset to monoparametric control the number of scrolls generated

**DOI:** 10.1016/j.dib.2020.105992

**Published:** 2020-07-06

**Authors:** J.L. Echenausía-Monroy, J.H. García-López, R. Jaimes-Reátegui, G. Huerta-Cuellar

**Affiliations:** a Dynamical Systems Laboratory, CULagos, Universidad de Guadalajara, Centro Universitario de los Lagos, Enrique Díaz de León 1144, Paseos de la Montaña, 47460, Lagos de Moreno, Jalisco, Mexico; b Applied Mathematics Division, Instituto Potosino de Investigación Científica y Tecnológica, IPICYT, Camino a la Presa San José 2055, Col. Lomas 4ta. Sección, 78216, San Luis Potosí, S. L. P., Mexico

**Keywords:** Chaotic system, Multi-scroll systems, Electronic implementation, Electronic Circuits, Nonlinear Dynamics, Chaos

## Abstract

The increasing usage of chaotic systems in the development of security systems, from mobile surveillance devices to the implementation of secure communication systems, leads to devising analog electronic implementations of research. This article presents the electronic implementation dataset from a multi-scroll chaotic system capable of generates until 9-scrolls throughout a monoparametric control based on a Saturated Non-Lineal Function (SNLF). The implemented system controls the number of generated scrolls being capable of producing attractors of single scroll and attractors with 3, 5, 7, and 9-scrolls. Moreover, the implemented system produces a family of bistable behaviors. The authors report the phenomenon in [1], where a complete analysis in the system has been carried out.

**Specifications Table**

**Table:** 

**Subject**	Statistical and Nonlinear Physics
**Specific subject area**	Nonlinear Dynamics, Chaos, Chaotic Systems, Multi-scroll Systems
**Type of data**	Figures
	Tables
	Schematic Circuit Design
	Experimental Time Series
**How data were acquired**	The data acquisition is developed employing a Data Acquisition Board (DAQ), NI USB-6363 to acquire the three state variables in the system.
	Two kinds of experiments were performed to analyze the dynamical system:
	a) Only modifying the bifurcation parameter, starting from random initial conditions in the electronic circuit.
	b) By modifying the initial conditions for each analyzed bifurcation parameter.
	The raw data contains 95,000 points for each state variable. The filtered data contains 65,000 points for each variable.
	Different control parameters are analyzed.
**Data format**	Raw
	Analyzed
	Filtered
	Time series are given in a plain text file
**Parameters for data collection**	The sampling frequency of 120 kS/s.
	Filtered using a Low-pass Butterworth filter order 3 with a cut-off frequency of 500 Hz.
	90,000 points for each temporal series.
	Resolution of 16 bits.
	Range of the ADC: Form −10 V to 10 V.
**Description of data collection**	Several control parameters (*α*) are analyzed, and for each control parameter, an exploration of the bifurcation parameter (*ρ*) is developed. For each ρ value analyzed, the initial conditions are changed utilizing a relay circuit to explore the whole system dynamics, where the time series from the three state variables are stored in a computer through a data acquisition board (DAQ). To ensure that the electronic system is evaluated with different initial conditions the charging and discharging times in the capacitors are calculated to activate and deactivate both data acquisition and switching of the voltage source through the relay circuit. The data acquisition and the digital pulse that controls the change in the bifurcation parameter are implemented by the DAQ board through a software application (Virtual Interface) developed in LabVIEW 12.
**Data source location**	Institution: University of Guadalajara, Centro Universitario de los Lagos (CULagos), Dynamical Systems Laboratory
	City/Town/Region: Lagos de Moreno, Jalisco
	Country: Mexico
**Data accessibility**	With the article
	Repository Name: Mendeley Data
	Data Identification Number: http://dx.doi.org/10.17632/yjypzzfrbt.2#file-873bb7d9–38e4–4ad9-b3df-6a0b1d0e1b4f
	Direct URL to Data: https://data.mendeley.com/datasets/yjypzzfrbt/2
**Related research article**	J.L. Echenausía-Monroy, J.H. García-López, R. Jaimes-Reátegui, G. Huerta-Cuellar, Parametric Control for Multiscroll Generation: Electronic Implementation and Equilibrium Analysis, Nonlinear Analysis: Hybrid Systems, *38*, 100,929, https://doi.org/10.1016/j.nahs.2020.100929.

**Value of the Data**

• The authors provide all the temporal series from an electronic implementation, where different kinds of behaviors are obtained, from several monostable attractors as well as the coexistence of single-scroll attractors.
• Datasets allow the use of the experimental behavior for further studies, as studies in synchronization, the generation of pseudo-random number generators, mobile surveillance devices, perturbation in dynamical systems, or only the dynamics (i.e., electroencephalography, and functional magnetic resonance).
• It is one of the very few cases where the generation of different multiscroll attractors are generated by modifying a single parameter and its implemented in electronics. Also, the system exhibits multistable behaviors, which potentiates its possible technological applications, increasing the value in the data presented.

## 1 Data description

**Fig. 1:**
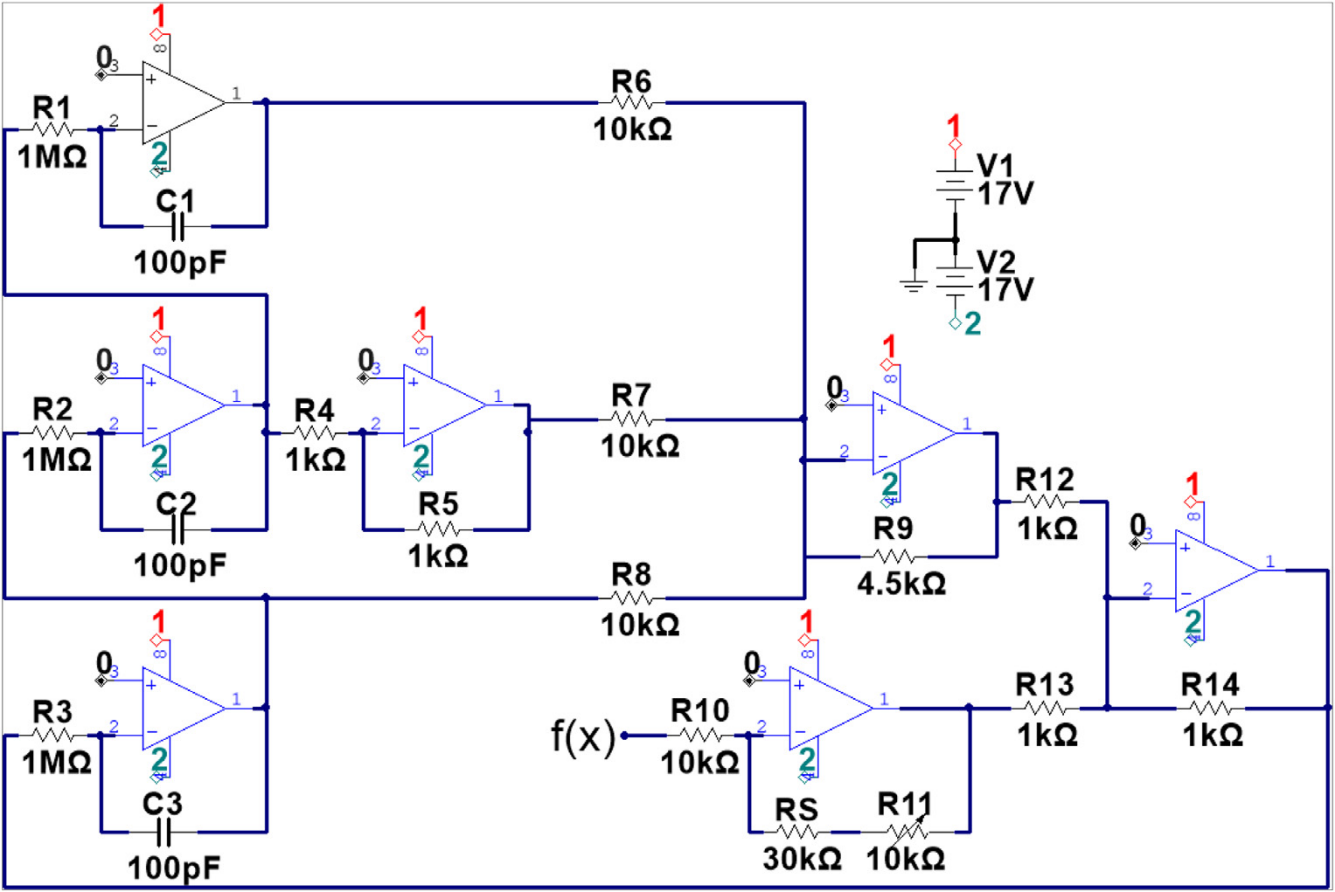
Circuit diagram of the linear operator from the implemented multi-scroll generator system. The elements values used for electronic implementation are described in the figure, and better described in [1].

**Fig. 2:**
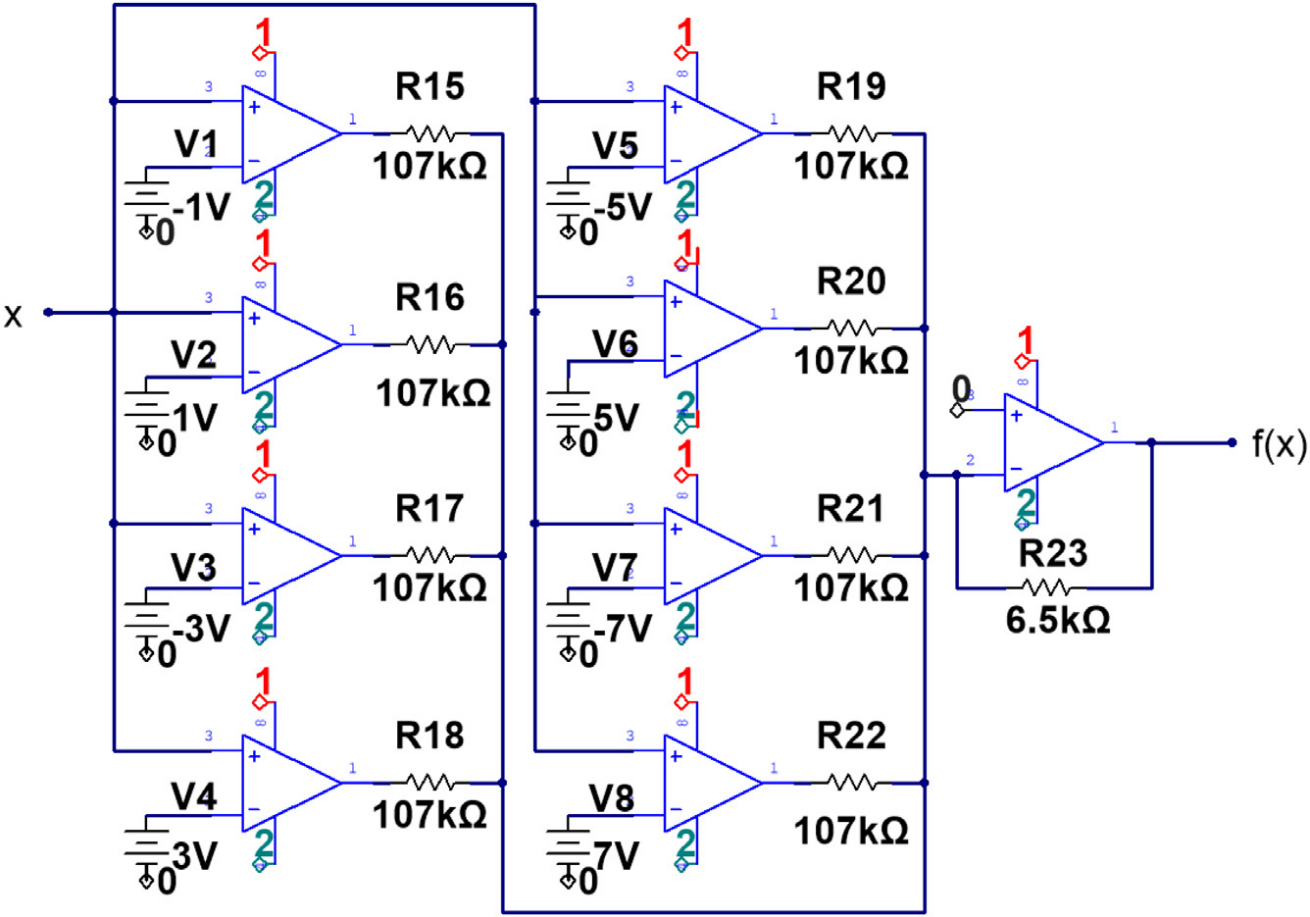
Circuit diagram of the Saturated Non-Lineal Function (SNLF) from the multi-scroll generator system implemented. The elements values used for electronic implementation are described in the figure, and better described in [1]. .

**Fig. 3:**
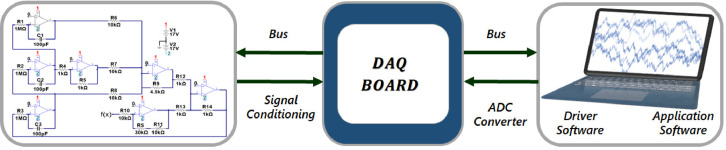
Experimental set-up. The change in the initial conditions and the digital pulse for the sweep in the *ρ* parameter is controlled utilizing a virtual interface.

**Fig. 4:**
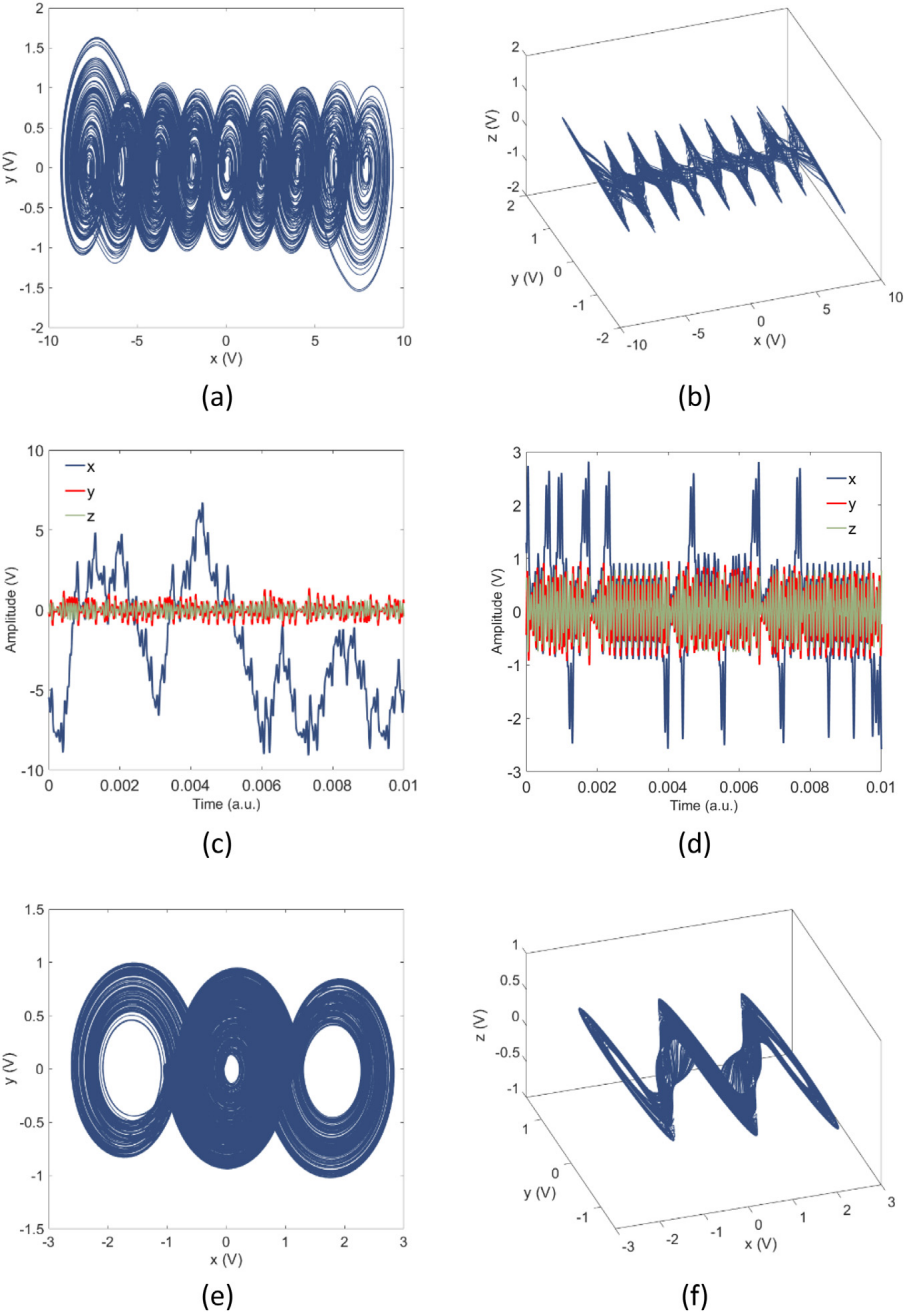
Experimental data. (a-c) Obtained for α=0.45,ρ=0.45. (d-e) Obtained for α=0.45,ρ=0.21.

**Fig. 5:**
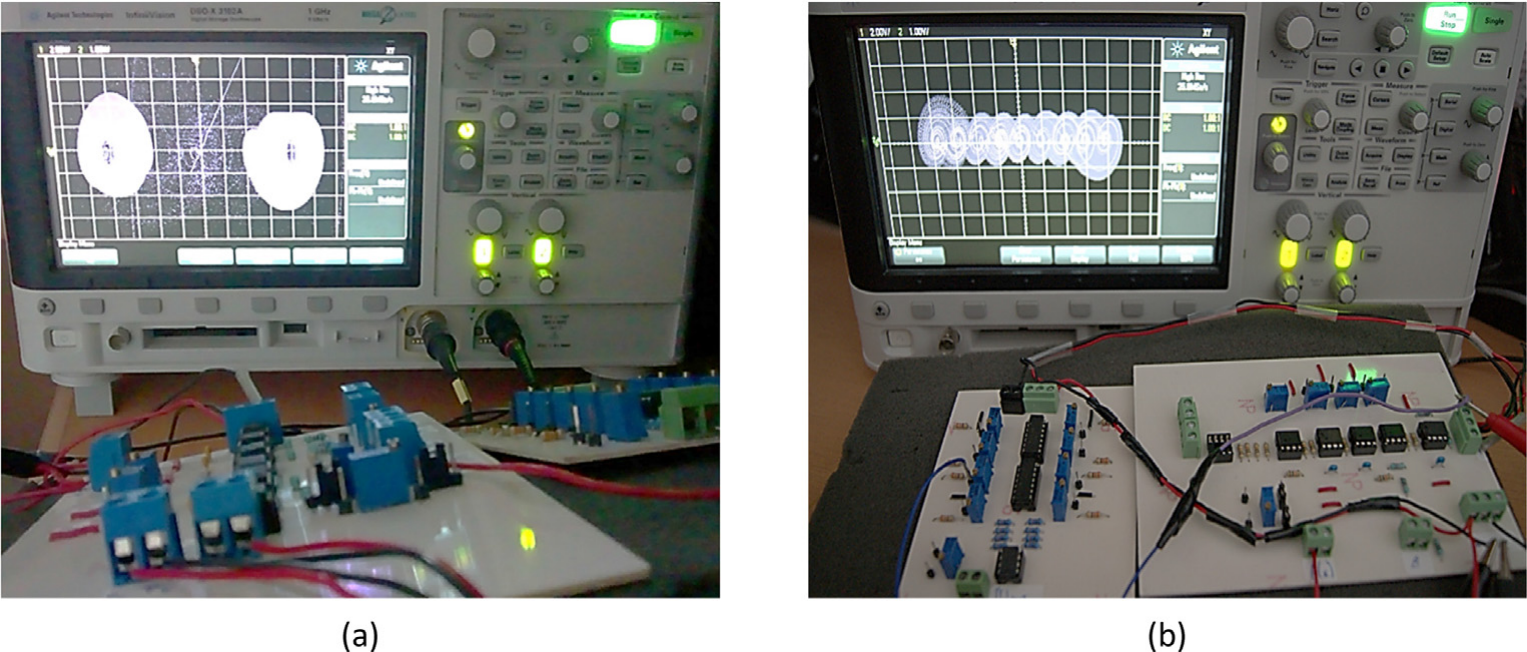
Attractors generated through the electronic implementation for α=0.45. (a) Bistable attractors associated to ρ=0.485, captured by means of changing the initial conditions and maintaining an infinite persistence in an *Agilent DSO-X 3102A* oscilloscope. (b) Natural dynamics in the electronic implementation, where a 9-scrolls attractor is observed in a digital oscilloscope.

The data files contain the temporal behavior form a multi-scroll chaotic circuit inspired on the jerk equation. Each temporal series contains the behavior of the three state variables. Each file contains three variables, numerically defined (TS_0_bif_0_cci_0.dat). The first numerical value corresponds to the experiment number, where the bifurcation parameter is only increased. The second numerical value corresponds to the present bifurcation parameter(*ρ*), which indicates the number of step used in the digital potentiometer (0–99) which reaches the 100 steps, and the last value indicates the changes in the initial conditions. The electronic circuit analyzed is shown in Fig. 1, Fig. 2. The corresponding experimental set-up for data acquisition is shown in Fig. 3. Some examples of temporal behaviors contained in the dataset are depicted in Fig. 4, Fig. 5.

## 2 Experimental design, materials and methods

The electronic diagram, shown in Fig. 1, corresponds to the implementation of the linear operator of the system and is designed in such a way that allows the exploration of all the *α* values defined for the UDS I region. The non-linear function corresponds to a Saturated Non-Linear Function (SNLF) for the generation of a 9-scroll attractor, depicted in Fig. 2. The electronic implementation of the system bifurcation parameter *ρ* is developed through the use of a 100kΩ trimpot potentiometer (*R_S_* in Fig. 1), and a 10 kΩ digital potentiometer (*R*_11_), to build the corresponding bifurcation diagram of the system and fully explore the dynamics generated by it. To achieve a high resolution in the bifurcation parameter (*ρ*), *R_S_* acts as an adding constant to *R*_11_, which means that to explore a range defined as 0.3 < *ρ* < 0.4, *R_S_*=30kΩ, and *R*_11_ varies in a ${\Delta_{R}}_{11}=$100Ω.

The automation scheme implemented for the data collection consists of exploring the entire range of values in the bifurcation parameter (*ρ*), through the use of a digital potentiometer for a fixed *α* value (scheme depicted in Fig. 3). For each *ρ* analyzed value, the initial conditions are changed through a relay circuit to explore the whole system dynamics, where the time series from the three state variables are stored in a computer through a data acquisition board (DAQ). The DAQ board used for this purpose is an *NI USB 6353* [2]. Unlike the numerical, where there is independent control over the three initial conditions of the state variables, in the electronic implementation, the three initial conditions are randomly modified at the same time. To ensure that the electronic system is evaluated with different initial conditions the charging and discharging times in the capacitors are calculated to activate and deactivate both data acquisition and switching of the voltage source through the relay circuit. The data acquisition and the digital pulse that controls the change in the bifurcation parameter are implemented in the DAQ board through a software application (Virtual Interface) developed in LabVIEW 12.

The temporal series stored in the computer is labeled as “TS_0_bif_0_cci_0.dat”. The first numerical value corresponds to the experiment number, where the bifurcation parameter is only increased. The second numerical value corresponds to the bifurcation parameter (*ρ*), which indicates the number of steps used in the digital potentiometer (0–99) which reaches the 100 steps, and the last value indicates the changes in the initial conditions.

In Fig. 4, two deferment behaviors exhibited in the dataset are presented, where an attractor and their corresponding temporal series, with 9-scrolls, are presented, when α=0.45,ρ=0.45, for the file named “TS_0_bif_0_cci_3.dat”, included in the folder Raw_Data, are shown in Fig. 4(a-c). In the same way, the dynamical behavior for α=0.45,ρ=0.21 is presented, corresponding to the file named “TS_2_bif_75_cci_5.dat”. Data files contain the three states variables of the electronic circuit, arranged in columns with a length of 95,000 elements. For a better description of the mathematical model that describes the multi-scroll generator, as well as a further analysis in the phenomenon that controls the generation of attractors with a different number of scrolls, consult [1].

An example of the circuit behavior where the coexistence of single-scroll attractors is presented with the files named “TS_287cci_7.dat”, and “TS_287cci_4.dat”, contained in the filtered data (α=0.45,ρ=0.485). Such behaviors are depicted in Fig. 5(a), which has been captured by means of changing the initial conditions and infinite persistence in an *Agilent DSO-X 3102A* oscilloscope. In Fig. 5(b) the natural dynamcs of the system, a 9-scroll attractor presented in Fig. 4(a), captured in an *Agilent DSO-X 3102A* oscilloscope is presented.

### 2.1 Multiscroll generator

The dynamical system implemented in the electronic circuit is governed by the set of equations shown in Eq. (1), better described in [1,[3], [4], [5], [6]].

$$\dot{x}=y,$$

$$\dot{y}=z,$$

(1) $$\dot{z}=-\alpha \left[x+y+z\right]+\rho \;f\left(x;k,h,p,q\right).$$

The parameter *ρ* controls the generation of attractors with a different number of scrolls, meanwhile, the *α* value is a control parameter associated with the stability of the equilibrium points in the system. Since it is locking for hyperbolic-saddle-node equilibrium points [3], [4], [5], [6], the control parameter is delimited by *α* ∈ (0, 1).

The non-linear function implemented (*f*(*x*)), whose purpose is to control the visit to the different domains, is delimited by the switching surfaces contained in the system, achieving this through the coexistence of a large number of one-spiral trajectories, responds as a Saturated Non-Lineal Function, better described in [3], and displayed in Eq. (2).

(2) $$f\left(x\right)=\sum_{m=p}^{q}f_{m}\left(x;k,h,p,q\right),$$

where *k* > 0 the slope of the series of functions, and *h* > 2 the delay in the saturated function, defined by the speed of the operational amplifiers; *p* and *q* are positive integers, m=1,2,…,n, that defines the number of scrolls to generate, being *n* the maximum of them.

## Ethics Statement

The authors certify that the manuscript *“Electronic Implementation Dataset to Monoparametric Control the Number of Scrolls Generated”* does not contain any studies with human or animal subjects.

## Declaration of Competing Interest

The authors declare that they have no known competing for financial interests or personal relationships which have, or could be perceived to have, influenced the work reported in this article.
